# Transparent PDMS Bioreactors for the Fabrication and Analysis of Multi-Layer Pre-vascularized Hydrogels Under Continuous Perfusion

**DOI:** 10.3389/fbioe.2020.568934

**Published:** 2020-12-23

**Authors:** Juan Liu, Huaiyuan Zheng, Xinyi Dai, Patrina S. P. Poh, Hans-Günther Machens, Arndt F. Schilling

**Affiliations:** ^1^Department of Plastic Surgery, The Central Hospital of Wuhan, Tongji Medical College, Huazhong University of Science and Technology, Wuhan, China; ^2^Clinic for Trauma Surgery, Orthopedics and Plastic Surgery, University Medical Center Göttingen, Göttingen, Germany; ^3^Department of Hand Surgery, Wuhan Union Hospital, Tongji Medical College, Huazhong University of Science and Technology, Wuhan, China; ^4^Department of Plastic and Reconstructive Surgery, Shanghai 9th People’s Hospital, Shanghai, China; ^5^Julius Wolff Institut, Charité - Universitätsmedizin, Berlin, Germany; ^6^Department of Hand Surgery and Plastic Surgery, Klinikum rechts der Isar, Technical University of Munich, Munich, Germany

**Keywords:** tissue engineering, vascularized hydrogels, PDMS, bioreactor, 3D printing

## Abstract

Tissue engineering in combination with stem cell technology has the potential to revolutionize human healthcare. It aims at the generation of artificial tissues that can mimic the original with complex functions for medical applications. However, even the best current designs are limited in size, if the transport of nutrients and oxygen to the cells and the removal of cellular metabolites waste is mainly dependent on passive diffusion. Incorporation of functional biomimetic vasculature within tissue engineered constructs can overcome this shortcoming. Here, we developed a novel strategy using 3D printing and injection molding technology to customize multilayer hydrogel constructs with pre-vascularized structures in transparent Polydimethysiloxane (PDMS) bioreactors. These bioreactors can be directly connected to continuous perfusion systems without complicated construct assembling. Mimicking natural layer-structures of vascular walls, multilayer vessel constructs were fabricated with cell-laden fibrin and collagen gels, respectively. The multilayer design allows functional organization of multiple cell types, i.e., mesenchymal stem cells (MSCs) in outer layer, human umbilical vein endothelial cells (HUVECs) the inner layer and smooth muscle cells in between MSCs and HUVECs layers. Multiplex layers with different cell types showed clear boundaries and growth along the hydrogel layers. This work demonstrates a rapid, cost-effective, and practical method to fabricate customized 3D-multilayer vascular models. It allows precise design of parameters like length, thickness, diameter of lumens and the whole vessel constructs resembling the natural tissue in detail without the need of sophisticated skills or equipment. The ready-to-use bioreactor with hydrogel constructs could be used for biomedical applications including pre-vascularization for transplantable engineered tissue or studies of vascular biology.

## Introduction

The aim of tissue engineering is to generate artificial tissues or organs with complex functions for replacement therapy, disease modeling and drug screening through a combination of desired cell types, bioactive molecules and biomaterials (Biondi et al., 2008; Khademhosseini et al., 2009; Atala et al., 2012; Abaci et al., 2016).

In various types of engineered tissue constructs, cell adhesion, proliferation, differentiation as well as extracellular matrix production has been studied (ECM; Agarwal et al., 2017; Phan et al., 2017). However, most of these constructs share a size limitation rooted in the metabolic needs of the embedded cells (Novosel et al., 2011). Cell survival is usually restricted within a limited range of 150–200 μm from the nearest capillary. Only in this area, the cells receive sufficient supply including oxygen, nutrition, and growth factors (Kannan et al., 2005). In bulky constructs lacking a vascular network, cell death often occurs in deeper areas resulting in tissue necrosis (Carmeliet and Jain, 2000). Moreover, not only do the blood vessels nourish the tissue, but they also allow disposal of the waste products of the cells metabolism (Kannan et al., 2005; Oh et al., 2017). Therefore, for the generation of functional tissues in clinically relevant sizes, inclusion of a functional volumetric and biomimetic vasculature is mandatory.

Several different approaches have been pursued to integrate vascular structure into engineered tissue constructs (Liu J. et al., 2015). The proposed methods include induction of vascularization in biocompatible hydrogels such as fibrin, collagen, and gelatin (Moya et al., 2013; Kim et al., 2015), integration of angiogenetic factors to form capillary networks in heterotypic tissue constructs (Chiu et al., 2012; Sekine et al., 2013), or creation of vascular structures by adhesion of ECs to the surface of channels in hydrogels (Miller et al., 2012; Kageyama et al., 2014). However, *in vivo*, tissues consist of a multitude of different cells usually arranged in layers, supplied by a common multilayered vascular structure comprising different types of specialized cells [e.g., endothelial cells, fibroblasts, smooth muscle cells (SMCs)] (Takebe et al., 2012; Peloso et al., 2015; Zakharova et al., 2017). To engineer such a structure for either *in vitro* study or for complex tissue engineering, the challenge is to be able to create similar layers with customized sizes and shapes with multiple different cell types.

In addition, it is necessary to design an appropriate 3D bioreactor for cultivation of these 3D vascularized constructs with stable and continuous perfusion. Generally, bioreactor systems are often used to perfuse culture medium through porous scaffolds so as to maintain cell viability throughout the constructs (Cartmell et al., 2003). For a functional vasculature culture *ex vivo*, many perfusion bioreactors have been developed to transport nutrients throughout tissue-engineered scaffolds of several tissue types, such as bone, adipose, and cardiac tissues (Radisic et al., 2004; Zhao and Ma, 2005; Grayson et al., 2008). However, in those studies, the sizes of vasculature as well as bioreactors could not be individually designed, which might limit their application in engineering larger and heterogeneous tissue constructs.

Polydimethysiloxane (PDMS) is an elastomer that contributed to the accessibility of microfluidics in academia and industry by enabling a straightforward and fast process of prototyping (Shiroma et al., 2016). It is low-cost, optically transparent, and able to integrate functional units such as valves and pumps, contributing to the establishment of lab-on-a-chip platforms (Unger et al., 2000; Nge et al., 2013). Moreover, PDMS exhibits outstanding gas permeability, which is important for biological experiments using living organisms or cells (Ren and Zare, 2012; Mu et al., 2013).

3D printing and additive manufacturing is one of the hottest recent advancements in designing and manufacturing for scientific research (Kivitz et al., 2013; Gao et al., 2014). It has been facilitating fabrication of bioreactors, implants and scaffolds with customized design on demand for tissue engineering and drug delivery (Chia and Wu, 2015). This technology has been widely accepted as a cost-effective tool providing the possibility of creating a variety of novel options. In our previous work, 3D printing was shown to provide an excellent tool to fabricate pre-designed molds and vascular patterns for tissue engineering with user-defined architecture (Ventola, 2014).

Herein, we report a novel PDMS bioreactor system that enables creating customizable multilayer, multi-cell type hydrogel constructs with pre-vascularized structures and perfusion of pre-vascularized channels. The approach uses 3D printing technology for designing and assembling the customized bioreactors and injection-molding for vascular structure fabrication. Our results suggest that (1) multicellular hydrogel constructs with vessel-mimicking structure can be fabricated *in situ* within customized PDMS bioreactors and (2) the constructs in this system can maintain multilayer structure under continuous perfusion in culture. The bioreactor system developed for this study establishes a novel *ex vivo* platform for incorporation of vascular networks into engineered tissue constructs.

## Materials and Methods

### PDMS Bioreactor Design and Fabrication

**FIGURE 1:**
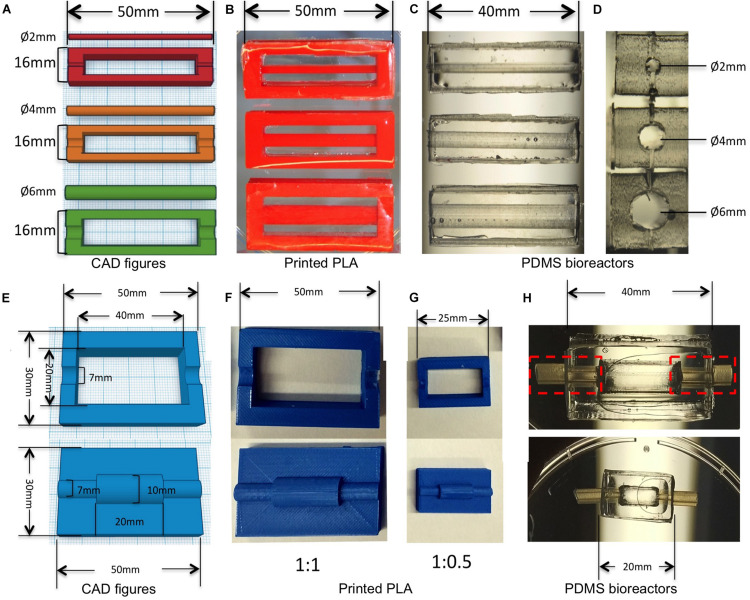
3D design and printed molds for creating bioreactors for different diameters of vessels. **(A)** CAD figures of molds for creating PDMS bioreactors with different inner chambers. **(B)** PLA molds printed with the design in panel **(A)**. **(C,D)** PDMS bioreactors with different sizes of inner chambers made from panel **(B)**. **(E)** CAD figures of molds for creating PDMS bioreactors with silicone tube connection. **(F)** PLA molds printed with the design in panel **(E)**. **(G)** PLA molds printed with the half size of panel **(F)**. **(H)** PDMS bioreactors with different length and diameter of inner chambers made from panels **(F,G)**.

We chose PDMS, a silicone-based polymer as a main component of our bioreactor technology for its excellent processability and biocompatibility. It can be easily casted, sterilized by autoclaving, is permeable to oxygen, and is transparent for real-time observation (Friend and Yeo, 2010; Liu et al., 2016). The bioreactors were designed with several parts and molds including culture chamber and inserts through the open access software Tinkercad^1^ (Figures 1A,E). Briefly, molds (length = 50 mm, width = 16 mm, height = 6 mm) were created with a rectangular chamber (length = 40 mm, width = 12 mm, height = 6 mm) for the PDMS bioreactors with rectangular solids (Figure 1A). In the middle of the bilateral width, positive patterns with half cylinders of different diameters (2, 4, and 6 mm) were included for insert fixation. Inserts with different diameters (2, 4, and 6 mm) were designed accordingly (Figure 1A). The sizes of the PDMS bioreactors could be modified customized by changing the size of the rectangular chamber in the molds (length = 40 mm, width = 20 mm for Figure 1F, length = 20 mm, width = 10 mm for Figure 1G). For better connection with the pumping system, two semi-cylindrical positive patterns with a diameter of 7 mm were designed for silicone tube assembly in later procedures (Figures 1E–H).

All of the molds were printed with polylactic acid (PLA) filament 3.0 mm (RepRap,Germany) using a low cost FDM printer (Ultimaker2, Ultimaker), with printing parameters: 100% infill, layer thickness = 0.6 mm; extruder temperature = 200°C, extrusion speed = 50 mm/s.

**FIGURE 2:**
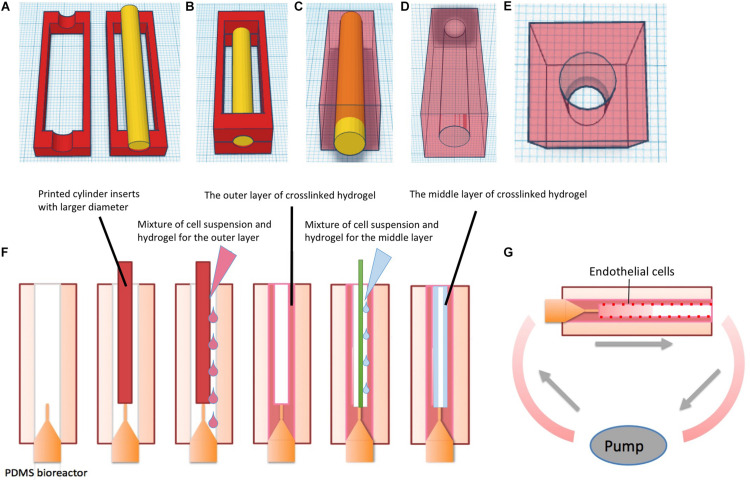
Schematic of fabricating PDMS bioreactor and multi-layer vessel structure in hydrogel constructs in PDMS bioreactors. **(A)** 3D design and printing of molds. **(B)** Assembling of the molds and the insert for chamber creation, the mixture of silicone base and curing agent was added into the surrounding space of the insert. **(C)** After curing time, the outer molds were removed from PDMS. **(D)** Gross-view of the PDMS bioreactor1. **(E)** Gross-view of the PDMS bioreactor2. **(F)** Fabricating single vessel with multilayer hydrogels via molding technique in PDMS bioreactors. **(G)** Culture of hydrogels with multi-layer vessel structure in PDMS bioreactor under continuous perfusion.

Printed PLA molds were assembled as shown in Figures 2A,B. Half-bioreactors were created against molds by soft lithography. PDMS Sylgard 184 (Dow corning, United States) was prepared with 10:1 ratio of silicone base to curing agent according to the instructions of the manufacturer. After being completely mixed, air bubbles were removed by being left at room temperature for 30 min. Then, mixtures were poured into the PLA mold. After 48 h at room temperature, cured PDMS elements were removed from the molds and the chamber was tightly assembled by combining 2-half parts of the bioreactor with silicone tubing and sealed by freshly prepared PDMS (Figures 1H, 2C–E. Finally, the complete PDMS bioreactors were washed with tap water and autoclaved at 120°C for 20 min.

### Cell Isolation and Cell Culture

Human umbilical vein endothelial cells (HUVECs) were isolated from human umbilical cords according to the protocols described previously (Crampton et al., 2007). HUVECs were cultured in Endothelial Cell Growth Medium2 (ECGM2 + supplement, PromoCell) and kept in a 37°C incubator with 5% CO_2_ and 15–20% ambient oxygen. SMCs were isolated from human umbilical arteries following Ribeiro’s method (Ribeiro et al., 2010). SMCs were cultured in Smooth Muscle Cell Growth Medium 2 (PromoCell) in a 37°C incubator with 5% CO_2_ and ambient oxygen. Green fluorescent protein expressing bone marrow-derived mesenchymal stem cells (GFP-SCP1) were kindly provided by Prof. Matthias Schieker (Experimental Surgery and Regenerative Medicine, University of Munich, Germany). Cells were cultured in DMEM supplemented with 10% fetal bovine serum and 10% penicillin and streptomycin (Biochrome). The medium was changed every 2–3 days for each cell type, and cells were passaged after 4–7 days by detachment with 0.25% Trypsin-EDTA solution (Biochrome). HUVECs and SMCs of passage <5 were used for the experiments.

### Fabrication of Multi-Layer-Pre-vascularized Hydrogels Within PDMS Bioreactors

The schematic diagram of the fabrication process is shown in Figure 2F. Commercial fibrinogen/thrombin kits (Tisseel, Baxter) were utilized to prepare fibrin hydrogels. The stock solution of fibrinogen was diluted to 45 mg/ml and thrombin to 50 U/ml with phosphate buffered solution (PBS) (Biochrome). Rat-tail collagen type I was purchased from Ibidi Company with an original concentration of 5 mg/ml.

Polydimethysiloxane bioreactors were assembled with 18G blunt end needles and 3D printed inserts. To prepare 1 ml of gel, 450 μl of thrombin solution (50 U/ml) was mixed with 50 μl GFP-SCP1 cell suspension at a density of 10^6^ cells/ml, resulting in a total volume of 500 μl. Similarly, 450 μl of fibrinogen solution (45 mg/ml) was mixed with 50 μl GFP-SCP1 cell suspension. Each of the solutions was transferred to a 1 ml syringe, respectively. Two syringes were injected through a Y-shape connector simultaneously into the space surrounding the insert (cylindrical-shaped, Ø = 4 mm) in the PDMS bioreactor. For collagen preparation, 3 mg/ml neutralized collagen solution mixed with 10^6^ cells/ml suspension was prepared according to the manufacture’s instruction and stored on ice. Within 5 min, the mixture was transferred to the PDMS bioreactor by syringe injection. Then the hydrogel in the bioreactor was incubated in a humidified incubator at 37°C and 5% CO_2_ for complete gelation. After 30 min, the insert was gently removed from the bioreactor.

For creating a second hydrogel layer, the 4 mm insert was removed and replaced with a smaller insert (cylindrical-shaped, Ø = 2 mm) within the bioreactor (Figure 2F). Fibrin/cell or collagen/cell hydrogel mixture was prepared following above procedures and then injected into the circular gap between the 2 mm insert and the previously formed hydrogel. Finally, after removal of the 2 mm insert, a tube-shaped construct comprising two layers of hydrogel with a hollow channel was formed.

Adhesion and endothelialization of endothelial cells on the surface of vessel channels was achieved by rotational culture of cells based on (Tang et al., 2008). In short Huvecs were incubated with Mitotracker Red (Abcam) at concentration of 50 nM for 3 h prior to experiment. The cell suspension was prepared in Ecgm2 at a density of 5 × 10^6^ cells/ml. Then, the Huvecs suspension was gently injected into the hydrogel channel from the inlet. Luer-lock adapters were used to close the inlet and the outlet. The bioreactor with the hydrogels was monted on an axial rotator and incubated with the cell suspension. The rotational speed was set to 4 rpm to allow cell attachment to the surface, allowing cell attachment to the surface. Rotational cell culture was maintained for 3–4 h in a 37°C incubator with 5% Co_2_ and ambient oxygen.

Thereafter, the bioreactor was connected to an ibidi Pump System (Ibidi). To mimic natural vascular perfusion, the bioreactor was unidirectionally perfused with fresh medium (Dmem + Ecgm2 at 1: 1 ratio) with a flow rate of 10 ml/min at a pulsatile frequency of 60/min. The total volume of medium for perfusion was 15 ml, which was recirculated to the inlet. The oscillation of the individual 1 s pulses were 0.5 s 20 mbar (systolic) and 0.5 s 0 mbar (diastolic) (Figure 2G). Bioreactors and pumps were maintained in a humidified incubator at 37°C and 5% Co_2_. The medium in the syringes was replaced with fresh medium every 48 h.

### Fluorescence Microscopy at Different Time Points of Perfusion

At day 0, 3, and 7, and the hydrogel constructs were harvested from the bioreactors. The perfusion culture was repeated five times at each time point. Cross sections of hydrogel constructs, with 2 mm thickness were obtained with a blade and transferred into a 48-well plate. Five cross sections were prepared from each hydrogel sample. NucBlue^TM^ Live ReadyProbes^TM^ Reagent (Thermo Fisher Scientific) was added into each well with concentration of one drop per milliliter. After 15 min incubation time, fluorescent images were captured with fluorescence microscopy (Zeiss) and confocal laser scanning microscopy (CLSM) (Olympus).

## Results and Discussion

Native organs often consist of multiple types of cells and extracellular matrix that are integrated together in spatial patterns (e.g., the vascular and parenchymal compartments of a tissue) (Florencio-Silva et al., 2015; Nederveen et al., 2016; Ogawa et al., 2018). For examples, native skin tissue is composed of layers of keratinocytes in epidermis, fibroblasts forming networks in the dermis and melanocytes on the bottom of dermis (Ogawa et al., 2018); native bone consists of a large amount of osteocytes in the Haversian system, osteoblasts and osteoclasts in the periosteum (Florencio-Silva et al., 2015). All the layers of cells are nourished by complex vasculature in native tissues. Therefore, the ability to fabricate multilayered tissues with vascular networks in a volumetric scaffold could significantly aid the development of low cost lab-on-a-chip, organ-on-a-chip and human-on-a chip devices (Liu et al., 2017).

In this study, we applied widely available low-cost 3D printing technology in conjugation with soft lithography and molding to develop ready-to-use multilayered, multicellular tissues with embedded vasculature inside custom PDMS bioreactors. The multilayer-pre-vascularized construct within our system could be directly connected to continuous perfusion pumps without the need of additional manual assembly.

### 3D Fabrications of Molds and PDMS Bioreactors

**FIGURE 3:**
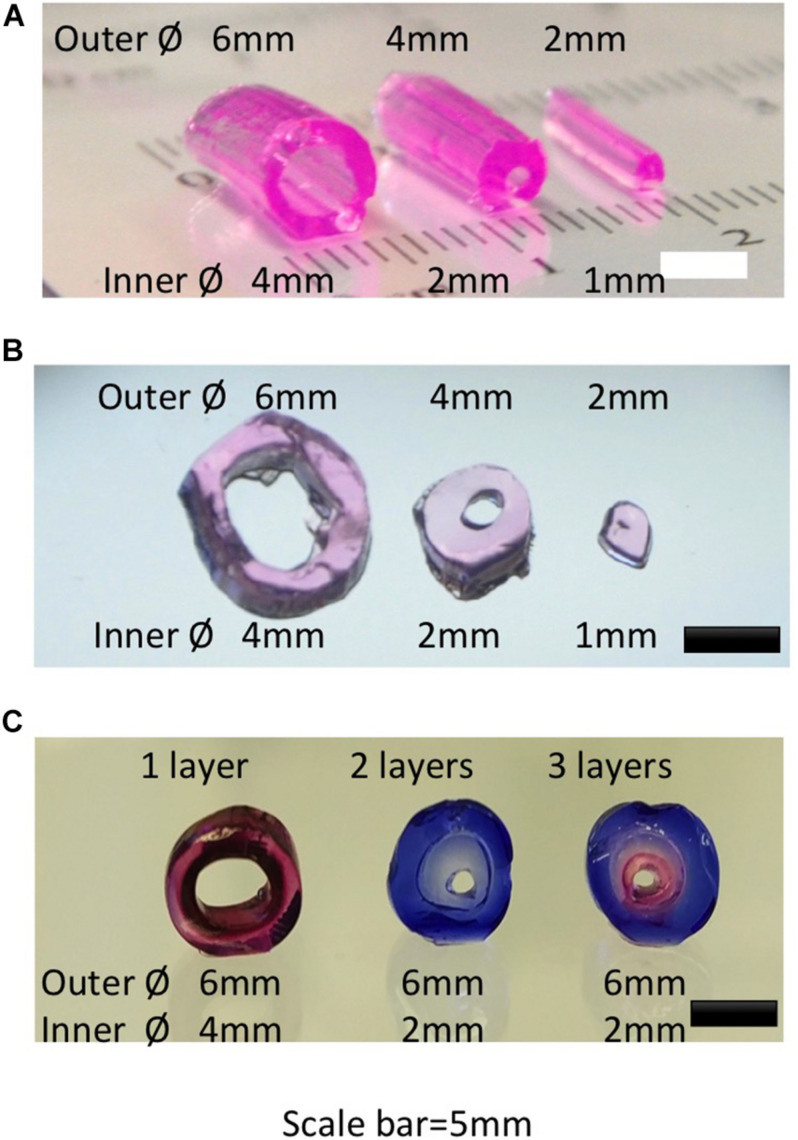
Gross view and microscopic view of hydrogel constructs created with different diameters and layers. **(A)** Gross view of single vessel hydrogels with different diameters. **(B)** Cross view of single vessel hydrogels in panel **(A)**. **(C)** Cross view of single vessel structure with different layers of hydrogels.

We designed and printed negative molds with PLA to create a transparent PDMS bioreactor for continuous perfusion with customized inner vascular architectures (Figures 1B,F,G; Liu, 2018). The inserts were created accurately with diameters of 2, 4, and 6 mm (Figure 1B). PDMS bioreactors were fabricated with corresponding inner chambers (diameter of 2, 4, and 6 mm) for hydrogel engineering (Figures 1C,D). The sizes and shapes of the inner chambers in the PDMS bioreactor were correspondent with the outer diameter of hydrogels created in later steps. For single vessel fabrication, cylindrical inserts with different diameter (1, 2, and 4 mm) were designed for the vascular channels (Figure 3A).

3D printing has already been widely used in laboratory research because of its flexibility to make customized geometries for various applications (Cartmell et al., 2003; Ventola, 2014; Chia and Wu, 2015). Using PDMS for bioreactor fabrication has the additional advantages of excellent processability (possibility to sterilize by autoclaving, flexible texture for casting, possible sealing), good biocompatibility, permeability to oxygen, and transparency for real-time observation (Figures 1C,H; Lee et al., 2003).

The geometries we have embedded in the design were accurately printed including not only the diameters of inserts, but also the lengths for the PDMS chambers (Figures 1C,H). The cylindrical inner chamber in this study was an original design created as a proof-of-principle example for single vessels, which has been shown pivotal to the architecture of multi-layer hydrogel constructs fabricated in subsequent procedures. The assembly process was performed in a sterile laminar hood after all parts had been autoclaved. The elasticity of the PDMS allowed a tightly sealed connection for perfusion using silicone tubes. We observed no leakage, suggesting it as a safer strategy compared to 3D-printed bioreactors made of materials such as PLA. Consequently, our bioreactor maintained sterility during perfusion and construct growth. These results collectively indicated that this combination of precise fabrication techniques and biomaterials not only provides high flexibility for the designs but also significantly minimizes the risk of contamination and improves the ease of handling.

### Multi-Layer-Pre-vascularized Hydrogels Within PDMS Bioreactors

The proteins of native extracellular matrix form hydrogels in most tissues, thus creating a hydrate microenvironment with good biocompatibility, which supports cell attachment, allows diffusion of oxygen and nutrients to cells, and is biodegradable (Geckil et al., 2010). Consequently, various types of hydrogels have been widely used as scaffolds in tissue engineering (Azab et al., 2006; Eyrich et al., 2007; Gillette et al., 2008; Sahoo et al., 2008; Ghanian et al., 2018). In this study, we successfully tested two different biological hydrogels (collagen and fibrin).

Fibrin gel is one of the natural polymers popularly used in surgery for the generation of a hemostatic niche for wound healing, as well as in laboratory research of tissue engineering (Jackson, 2001; Laurens et al., 2006; Ahmed et al., 2008). Fibrin hydrogel is prepared by mixing of fibrinogen and thrombin, leading to rapid *in situ* polymerization (Chapter IV, 1947) (Bailey and Bettelheim, 1955). It has excellent biocompatibility and non-toxic degradability allowing cell survival and proliferation, contains cell-binding sites for cell attachment, and an adjustable mechanical stiffness. This can be modified to create 3D porous structures for a variety of tissue engineering applications (Yasuda et al., 2010; Skaat et al., 2012). We first tried to only use fibrin and thrombin to generate the hydrogels (data not shown), however the polymerization in the y-shaped syringe was so fast, that we were unable to inject the gels with uniform cell distribution. Thus, we included Collagen type I into the solution. Collagen type I is a natural protein, widely used in tissue engineering and regenerative medicine (Pawelec et al., 2016). It has been involved in creating cell-free materials for retention and release of growth factors as well as for delivering cells to contribute to regeneration processes (Kim et al., 2016; Nakaegawa et al., 2016; Schuh et al., 2018).

At the used concentrations (3 mg/ml for collagen; 45 mg/ml of fibrinogen + 50 Ul/ml of thrombin), the Young’s modulus of the hydrogels was reported at 0.5 kPa (Duong et al., 2009) and 4 kPa (Raub et al., 2010), respectively. The stiffness of the hydrogels should be adjusted to the diameters of the vascular channels to avoid deformation or collapse. To create even stiffer tissues it could be feasible to combine soft hydrogels with stiffer synthetic polymers such as electrospun meshes (Brunelle et al., 2018).

In recent decades, a variety of methods has been reported for fabrication of vascular-like networks within hydrogels including molding, soft lithography, photocuring, 3D bioprinting and modular methods (Sadr et al., 2011; Miller et al., 2012; Liu J. et al., 2015; Massa et al., 2017; Zhang et al., 2017). For single vessel fabrication, molding was used in combination with syringe needles for channel creation. With this approach, the variety of sizes and lengths of the vessel structures was limited by the types of syringe needles (Kageyama et al., 2014). 3D printing on the other hand provides more flexibility of the design (Figures 2, 3). Following the molding process, hydrogel tubes with different outer and inner diameters were created. Resulting hydrogel tubes showed consistence with the 3D geometries as designed through Tinkercad (Figures 3A,B; Liu et al., 2016). Vessel structures were created with multiple layers (Figure 3C). This basic unit with a HUVEC-coated channel surrounded by a multilayer architecture with different cell types could be customized to fulfill the requirements of most volumetric tissue constructs. There may be new challenges, when the tissue is bigger. In order to construct an engineered tissue with the size mimicking native organs, the basic unit will be fabricated with endothelial layer, SMCs layers and functional cell layers from target organs, with the sizes ranged from millimeters to centimeters. The assembling of multiple basic units with graded sizes could provide a major vascular network for bigger organ engineering.

### Multilayer Cell Culture Under Continuous Perfusion

Continuous perfusion of medium through the bioreactor was accomplished and cells were encapsulated within the hydrogel matrices and were successfully cultured for at least 7 days. During the experiment, no leakage was observed from the inlet or outlet connectors.

Anatomically, a natural blood vessel is composed of a single layer of ECs at the inner surface (intima) and one (capillaries) or more additional layers consisting mainly of extracellular matrix, SMCs, fibroblasts, and mesenchymal stem cells (MSCs). The intima serves as a structural barrier between the circulation and surrounding tissues, and prevents platelet aggregation (Liu Y. et al., 2015). The medial layer (media) of the vessel wall is composed of SMCs embedded in elastic ECM which allow the vessel to react to pressure changes *in vivo* (Chaterji et al., 2010). The outer layer (adventitia) mainly consists of connective tissue and protects the vessel against mechanical forces.

**FIGURE 4:**
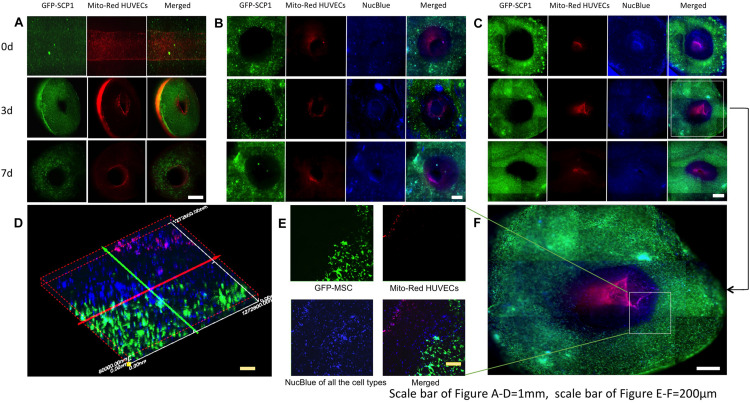
Fluorescence images of multilayer structures, mimicking vessel layers in the hydrogels at different time-points of perfusion. **(A)** Images of two-layer vessel structures after 0,3,7 days perfusion. **(B)** Images of three-layer vessel structures in fibrin hydrogels after 0,3,7 days perfusion. **(C)** Images of three-layer vessel structures in collagen hydrogels after 0,3,7 days perfusion. **(D)** Vessel structure formation and cell growth in collagen hydrogels after 3-day perfusion. **(E)** Confocal microscopic image of three-layer structure in the hydrogels. **(F)** 3D reconstruction of three-layer structure in panel **(E)**.

In this study, 2-layer and 3-layer hydrogel constructs composed of ECs/MSCs and ECs/SMCs/MSCs were tested respectively (Ventola, 2014). HUVECs were tracked using Mitotracker Red (Mito-Red), MSCs were modified by GFP, with all the live cell nuclei tracked with NucBlue in 3-layer constructs. This simple tracking technique allows direct observation of multilayer structures of the cross-section of samples without fixation and immunostaining procedures. In the 2-layer hydrogel culture system, within the fibrin matrix, GFP-SCP1 cells were distributed uniformly within the hydrogels and endothelial cells were coated on the inner surface of the channels (Figure 4A). After 3 days of perfusion culture, the 2-layer structure was well maintained and cells grew with a good viability within the hydrogels (Figure 4A). The endothelial layer was maintained for at least 7 days. For the 3-layer hydrogel system, in the outer layer GFP-MSCs (SCP-1) were embedded, the middle layer consisted of SMCs and the inner layer was endothelialized by mitotracked HUVECs. All living cells were tracked using NucBlue, a fluorescent dye which specifically binds to the nucleus of live cells (Figures 4B–F). A 3-layer hydrogel system was created with obvious boundaries (Figures 4D–F). The cell sheet organization was maintained after both 3 and 7 days of perfusion culture. CLSM images clearly show three layers of differently composed sheets as well as multiple types of cells mimicking a typical vascular structure. Cell growth and network formation among cells could be observed (Figures 4D–F). Further research will be necessary to establish the functional integrity of the different layers (CD31, CD144 expression, barrier fuction, patency) *in vitro* and *in vivo*.

Pilot experiments had shown that at pressures <20 mbar, channels with a diameter of 1 mm would sometimes collapse, while at 20 mbar all channels stayed open throughout the experiment. The pulsatile frequency was set to mimick the pulse in native tissue (60 pulses per minute) and the systolic pressure here was the minimum value allowing successful perfusion through the channel in hydrogels. The flow rate was set as 10 ml/min in the software of pump. The shear stress that endothelial cells would be exposed to depends on the viscosity of perfused medium, the length and diameter of channels the flow rate as well as the pressure drop during perfusion. The wall shear stress in a rectangular microchannel can be calculated. Based on the equations used in Ravin et al. (2012) the shear stress that endothelial cells could be exposed to our 2 mm channels could be estimated at 18.75 dyn/cm^2^/10 s, which is similar to the value in other microfluidic studies on cell growth and proliferation (11.5–23 dyn/cm^2^) (Maneshi et al., 2017). It might be interesting to study the correlation of shear stress with the function of endothelial cells in different channel geometries.

In this study, we terminated the perfusion after 7 days to evaluate the feasibility of PDMS system for fabricating pre-vascularized hydrogels and consequent perfusion. Longer-term perfusion led to shrinking of the hydrogels. While the inner channel stayed stably connected to the perfusion system, the outer layer of the hydrogels then detached from the PDMS chamber, which resulted in the scaffolds floating during perfusion with a gap between the scaffold and the reactor wall.

Typically, hydrogels left to themselves in contact with fluid rather tend to increase their volume according to their swelling ratio (Lu et al., 2018). We assume that the constant perfusion of the hydrogels with 10 ml/min should provide enough fluid to the gels to replace possible loss due to evaporation as we did not observe any obvious leakage of our bioreactors. Moreover, the shrinkage of scaffolds could already be observed at 7 days, when scaffolds were still completely surrounded by the medium. Therefore, we assume that this shrinkage is related to known cellular high traction forces exerted by the cells on the matrix as well as hydrogel dissolution after long-term culture but not dehydration (Lu et al., 2018), which would point at a healthy activity of the cells in our experiment.

It is reported that degradable peptides enabled entrapped cells to rearrange their cytoskeletal structure (Khetan et al., 2013). Traction force microscopy showed displacements of embedded tracker particles resulting from cell-generated forces in the degradable hydrogels. It might be possible to counter this effect by using stiffer hydrogels or soft hydrogels combined with embedded stiff polymers to enable perfusion of vascularized hydrogels for longer time spans.

In Figure 4B, the samples were prepared to 2 mm thickness with blades and observed with fluorescent microscopy, therefore, the images were not taken in exactly one focal plane but as an overlap image with a certain thickness, with differences of visible cell numbers in those figures. As the crosslinking of collagen was much slower than fibrin, the mixture of the cell suspension with hydrogels was probably more efficient in collagen. That might be the reason, why the cells were distributed more uniformly in collagen scaffolds. Furthermore, the different cell distribution in fibrin and collagen hydrogels (Figures 4B,C) suggests a role of the extracellular matrix in this. Thus, the uniformity of the cell distribution may also be influenced by the uniformity of the used hydrogel.

In previous studies, various approaches have been developed to fabricate multilayer vascular tissues, including rolling of cell sheets, assembling of toroidal cell aggregates as well as bioprinting with cell-laden hydrogels (Yang et al., 2007; Masuda et al., 2012; Lee et al., 2014). However, formation of the monolayer of endothelial cells is challenging when perfusion culture is not provided. Using highly flexible 3D printing in combination with injection molding, we show here the practical design and successful production of hierarchical, multilayered, and thick constructs mimicking the tissue structures of capillary or larger blood vessels. This open technology can be easily adapted according to individual research goals.

However, there are still some limitations of this study, which will be investigated in the future work. Engineering native tissue is not only based on principles of cellular and molecular developmental biology and morphogenesis, it is very much guided by bioengineering and biomechanics. Therefore, the relationship between cell behavior and mechanical properties such as stiffness, flow rates, distinct and dynamic loading conditions, uniformity of hydrogels need to be studied. The functionality of the endothelialized channel as well as surrounding cells has not been demonstrated in this study and further work should be focused more on biological characteristics of cells. Moreover, the composition of biomaterials might be modified so as to guarantee longer-term (>4 weeks) cultivation of multilayer cell-laden hydrogels surrounding vascularized channels based on this methodology.

## Applications

The methods presented here provide a new strategy for building multilayer 3D hydrogel constructs with customized vasculature *in vitro*. Our proposed methods on vascular network fabrication are efficient, simple, reproducible, and economical. By selecting the relevant cell types and matrix components it can theoretically be applied for construction of nearly every tissue such as skin, muscle, vessel, cartilage, trachea, or lymphatic duct engineering to name just a few possibilities. The technology allows the construction of larger-scale 3D tissues, for study of physiological/pathological processes like spatiotemporal cell migration, cell differentiation and clot formation, body-on-a-chip systems, or evaluating drugs and toxins in vascular-like environments *in vitro*. The transparency of PDMS device provides the possibility of real-time screening of these processes.

## Conclusion and Perspectives

The possibility to fabricate multilayer 3D vascularized microfluidic hydrogel units is pivotal for the development of tissues on a chip. In this study, we demonstrate how 3D printing-based technology allows the formation of multi-layer vascular structures within cell-laden hydrogels as well as customized PDMS bioreactors for continuous perfusion and real-time observation. This approach enables fabrication of hydrogel-based multilayer vascular devices in a cost-effective and fast manner, which has a huge potential for viable 3D cell culture, complex tissue engineering, disease modeling as well as drug screening.

This model can be applied to mimic natural tissue architecture and create bigger and larger multilayer hydrogel constructs with corresponding layers of functional cells to study cell morphology, differentiation, and intercellular interaction in engineered tissue constructs. Moreover, cell populations and biological function within the prevascularized hydrogels are influenced not only by the flow rates and pressure drops from the PDMS device, but also by the interaction between cells and biomaterials. This is an interesting topic, that might be investigated as a new project. Further optimization of the hydrogel composition in each layer, cell selection, cell density and perfusion parameters may enable the preparation of 3D vascular tissues mimicking natural functions for various biomedical applications.

## Data Availability Statement

The original contributions presented in the study are included in the article/supplementary materials, further inquiries can be directed to the corresponding author/s.

## Author Contributions

JL and HZ designed and carried out the study. JL and XD wrote the manuscript. XD and PP provided the technical support for the study. H-GM provided proofreading for this manuscript. AS was the total instructor for this study. All authors contributed to the article and approved the submitted version.

## Conflict of Interest

The authors declare that the research was conducted in the absence of any commercial or financial relationships that could be construed as a potential conflict of interest.

## Footnotes

1 www.tinkercad.com
